# Memory and Language in Middle Childhood in Individuals with a History of Specific Language Impairment

**DOI:** 10.1371/journal.pone.0056314

**Published:** 2013-02-07

**Authors:** Anne Hesketh, Gina Conti-Ramsden

**Affiliations:** School of Psychological Sciences, The University of Manchester, Manchester, United Kingdom; University of British Columbia, Canada

## Abstract

This study reports on the sensitivity of sentence repetition as a marker of specific language impairment (SLI) in different subgroups of children in middle childhood and examines the role of memory and grammatical knowledge in the performance of children with and without language difficulties on this task. Eleven year old children, 197 with a history of SLI and 75 typically developing (TD) peers were administered sentence repetition, phonological short term memory (PSTM) and grammatical morphology tasks. Children with a history of SLI were divided into four subgroups: specific language impairment, non-specific language impairment, low cognition with resolved language and resolved. Performance on the sentence repetition task was significantly impaired in all four subgroups of children with a history of SLI when compared to their age peers. Regression analyses revealed grammatical knowledge was predictive of performance for TD children and children with a history of SLI. However, memory abilities were significantly predictive of sentence repetition task performance for children with a history of SLI only. Processes involved in sentence repetition are more taxing of PSTM for individuals with a history of SLI in middle childhood in a way that does not appear to be the case for TD children.

## Introduction

Children with specific language impairment (SLI) have traditionally been diagnosed by exclusionary criteria; i.e. language difficulties in the absence of significant motor, sensory or nonverbal cognitive limitations. However, there is accumulating evidence of non-linguistic deficits in their profile, particularly memory difficulties, and a recognition that these children’s pattern of difficulties may shift with maturation. There is also evidence of a strong genetic influence on the manifestation of SLI with a complex pattern of inheritance [1]. Nevertheless, there is a need to explore underlying cognitive or psycholinguistic ‘endophenotypes’ [2], both to strengthen genetic knowledge and to understand the functioning of children with the condition. Limitations on phonological short-term memory (PSTM) [3], difficulty using morphological affixes [4], an impairment of computational syntax [5], an auditory perceptual/processing deficit [6] and a generalised limitation of processing speed or central executive [7] have all been proposed as loci of impairment. Of these suggestions, general processing, PSTM and grammatical morphology have received particular attention. The most extensive body of research supports the difficulties children with SLI face with PSTM and with grammatical morphology, and provides evidence for the heritability of both factors, although they arise from separate genetic locations [2]. What is less clear is whether these types of difficulties predict progress in children with SLI at different stages of development.

### PSTM, morphological knowledge and language functioning in children with SLI

In an evolving model of working memory, Baddeley [8]–[9] sees PSTM as a domain-specific area for the temporary storage of verbal information. It reports to a domain-general central executive which oversees and coordinates aspects of attention and memory in cognitive tasks. Research into the memory capacity of children with SLI has consistently shown deficits which appear predominantly domain specific as fewer children with SLI appear to have difficulties with visuospatial memory tasks [10]. Gathercole and Baddeley [11] were among the first to demonstrate that nonword repetition was a marker of SLI as it discriminated between children with language disorder and either age or language matched typically developing (TD) controls. Nonword repetition has since been widely used in research with children with SLI. There is robust evidence that children with SLI have nonword repetition deficits at a group level, even in children with a history of language difficulty in whom overt symptoms have resolved [3] [12]–[15]. Sensitivity and specificity of the task is high when comparing groups of clinically identified children with SLI and their typically developing peers. However, the task is less discriminating in population studies [16] and it is also known that a small but notable proportion of children with SLI have intact nonword repetition skills [17].

Nonword repetition has been seen, until recently, as a relatively pure measure of PSTM compared, for example, to word repetition which is more directly influenced by lexical/semantic knowledge [18]. However, there is currently debate as to what other types of knowledge may influence performance on nonword tasks. There is evidence, for example, that children’s lexical and sublexical knowledge impact on children’s performance [19]. Children with SLI are likely to have limited knowledge and processing in this area, thus poor performance in nonword repetition tasks is likely to reflect lexical limitations as well as PSTM constraints. Arguably, lexical knowledge and PSTM are interdependent. What is important to point out is that although nonword tasks are likely to be influenced by other types of knowledge, there is agreement that it does tap, at least partly, PSTM abilities (see Coady & Evans [20] for a review).

Morphologically, English-speaking children with SLI have long been noted to have difficulty marking tense and agreement via the third person singular *–s*, copular or auxiliary *is/are*, and the past tense *–ed.* Use of finite verb morphology was found by Bedore and Leonard [21] to be sensitive and highly specific in identifying pre-school children with SLI, and their difficulties extend into the school years. Thus, linguistic morphological knowledge appears to be related to overall language functioning in children with SLI. It has been proposed that children with SLI see such marking as an optional rather than an obligatory feature of their language and that this phase of development lasts for much longer than in typically developing children; the Extended Optional Infinitive theory [22]. The coherence of this theory and the consideration of grammatical morphology as a marker of SLI are supported by demonstration of generalisation following intervention across morphemes with dissimilar surface forms [23].

More recently, there has been recognition of the value of sentence repetition (or sentence recall) as an indicator of language functioning in SLI. Of four potential markers investigated by Conti-Ramsden et al. [3] (the others being past tense provision, third person singular use and nonword repetition), sentence repetition showed the best combination of sensitivity and specificity in categorising children into SLI or TD groups, regardless of current level of language ability (i.e. even in children whose surface performance on language measures had resolved). Archibald and Joanisse [24] confirmed the value of sentence repetition as a marker of language impairment, combining high sensitivity and better specificity than nonword repetition.

There remains a lack of clarity as to the underlying impairment or impairments which lead to difficulty with language functioning as evidenced by sentence repetition difficulties. The task involves short term memory storage but there are additional linguistic factors, both grammatical and semantic, among others, which are also likely to contribute [25]. Riches et al. [26] showed a significant relationship between digit span and sentence repetition errors in children with language impairment, as well as a strong association between sentence repetition and nonword repetition, thus emphasising the contribution of memory to performance in this task. In contrast, Poll et al. [27] found that neither forward nor backward digit span significantly correlated with either nonword or sentence repetition in a group of adults with SLI, suggesting that sentence repetition performance is not purely a function of memory and also that it may vary at different stages of maturity. Conti-Ramsden et al. [3] suggest that sentence repetition performance is influenced by a number of factors including both short term memory and established language abilities such as grammatical morphology, both of which appeared impaired in their sample of children with SLI. Indeed, the multifactorial nature of the sentence repetition task, including demands on both memory and specific aspects of language, may be what makes it such a valuable marker [27]. The linguistic component of sentence repetition may either support performance of language-competent children who can make use of the added predictability of sentence content and form, or hinder children with language difficulties due to its demands on an area of impairment.

Sentence repetition is also a valuable marker of SLI because it does not correlate strongly with nonverbal abilities, i.e., performance IQ (PIQ). Researchers have pointed out that clinical markers of SLI should be largely independent of PIQ as one is interested in identifying the language deficits of individuals and not their general learning ability. Sentence repetition, as well as nonword repetition and grammatical morphology, have been found not to be correlated with PIQ in children with SLI [3] [13] [28] with values ranging from .16 to .24 and sentence repetition yielding the lowest correlations. This pattern has also been observed in TD children with values ranging from .01 to .24 ([13]; correlations based on data from [29]; [30]).

A picture is building of children with SLI being characterised, at least partly, by a combination of difficulties, some related to specific aspects of language, some related to phonological aspects of short term memory. There is also increasing evidence of the value of sentence repetition as a marker of SLI [24], even in children whose surface language performance has evolved to be within normal limits [3]. What needs further investigation is the extent to which memory difficulties in areas such as PSTM and specific linguistic problems such as those involving grammatical morphology, contribute to the ability of individuals with SLI to recall sentences.

### Aims of the present study

In this study, we focus on language functioning as measured by a widely acknowledged marker task, sentence repetition, and two predictor factors, PSTM (as measured by nonword repetition) and grammatical morphology (as measured by past tense affixation) both of which have themselves been suggested as markers for SLI [3] [12]–[14] [21]–[22].

Specifically, this study aimed firstly to examine the sensitivity of sentence repetition to different subgroups of children with a history of SLI and secondly, to investigate the role of PSTM abilities and morphological knowledge in sentence repetition in children with and without a history of SLI. With a few exceptions [3] [13], most of the evidence available regarding the predictor factors (PSTM and tense affixation) has been gathered in early childhood between the ages of 4 and 7 years [16] [31]. In contrast, the data in this study focuses on children at 11 years of age. It is important to examine middle childhood in order to increase our understanding of both the trajectory of SLI as a disorder and the potential influences the educational system may have on this. In particular, by the time children are 11 years of age they have engaged in a number of years of primary school education. For most children this age marks the end of primary schooling and the transition to secondary or high school education.

We were able to demonstrate that sentence repetition was sensitive as a marker of different subgroups of children with a history of SLI, regardless of whether they had current or resolved receptive language difficulties. In addition, the processes involved in language production were found to be taxing of PSTM for individuals with a history of SLI in middle childhood in a way that did not appear to apply to TD children of the same age. Memory abilities were a significant predictor of sentence repetition performance in individuals with a history of SLI.

## Methods

### Ethics Statement

Informed written consent was gained from parents or legal guardians on behalf of the child participants. Ethical approval for the study was obtained from the Senate Committee for the Ethics of Research on Human Beings, The University of Manchester, UK.

### Background of Participants with a History of SLI

An initial cohort of 242 children (24% female; 11% had exposure to languages other than English) with a history of SLI were recruited as part of the Manchester Language Study. These individuals represented a randomized sample of all 7-year-olds attending 50% or more of their school week in a language unit in England. Language units are classes that offer specialist language environments. The staff usually includes a specialist teacher and a classroom or speech-therapy assistant; regular speech and language therapy input is provided by a qualified therapist.

All known language units across England were contacted by telephone, and teachers were asked to report how many 7-year-old children were attending more than 50% of the school week. They were also asked about additional difficulties; children with known current hearing loss, major physical disabilities, definite diagnosis of autism or of moderate learning difficulties were excluded at this stage. Thus, no specific “SLI” criteria were used at selection. Rather, enrollment in a language unit and the absence of other diagnoses were used as identification of the sample. However, subsequent analysis showed that 84% of the sample met traditional discrepancy criteria for SLI. Approximately 38% had expressive only difficulties, 53% had difficulties in both expressive and receptive modalities, and 9% were thought by clinicians to have complex or pragmatic language difficulties [32]. Three children had poor nonverbal IQ (< 70 standard score) but were not thought to be globally delayed, hence the absence of diagnoses of moderate learning difficulties. Thus, the children participating in this study had not been selected a priori on the basis of having met criteria for SLI and they exhibited a variety of types and severity of language impairments. Full details of the initial cohort’s profiles of impairment can be found in [33].

At age 11, 197 children were available for follow-up assessment; 49 (25%) were girls, 24 (12%) had exposure to languages other than English, average age was 10;11 years (SD  =  5 months). There is increasing evidence to support the view that SLI is a developmental disorder that can change over time. Several studies have reported a decline in PIQ, relative to age expectations, from childhood to early adulthood, in individuals with a history of SLI [34]–[35]. Research from the Manchester Language Study has shown that nearly one third of individuals exhibit a slowing down in the growth of their nonverbal skills from childhood to adolescence [35]. These individuals with a history of SLI who in childhood had nonverbal skills within the normal range do not continue to do so later in development. For convenience, however, the 197 children participating in this study are collectively referred to as having a history of SLI whatever their current cognitive and language status.

### Subgroups of Children with a History of SLI

All the above participants had a history of SLI in the absence of significant cognitive motor or sensory difficulties but by age 11 the children’s abilities had evolved to different profiles. Given that recalling sentences was the focus of the study as a marker of SLI and this task involves expressive language abilities, children were divided according to their performance on a receptive language assessment task, the Test for Reception of Grammar (TROG [36]), and performance IQ (PIQ) tasks, the Block Design and Picture Completion subtests of the Wechsler Intelligence Scale for Children (WISC-III [37]). The TROG is a multiple-choice, pictorial test designed to assess understanding of grammatical constructions. The WISC-III subtests used require the child to copy small geometric designs with four or nine larger plastic cubes and to identify missing parts of pictures respectively. The scores on the two WISC-III subtests were combined to form an estimated PIQ. This procedure yielded four subgroups:

SLI - specific language impairment (TROG < 85, PIQ ≥ 85), N  =  32

NSLI - non-specific language impairment (TROG < 85, PIQ < 85), N  =  56

LIQRes - low cognition, resolved receptive language (TROG ≥ 85, PIQ < 85), N  =  34

Res – resolved receptive language (TROG ≥ 85, PIQ ≥ 85), N  =  75

The profile of test scores for each of the four subgroups of children with a history of SLI and their original scores at 7 years are presented in table 1.

**Table 1:** Receptive language and PIQ scores for subgrouping children with a history of SLI at 11 years and original scores at 7 years.

	TROG^a^		PIQ^a^	
	M (SD)		M (SD)	
SLI subgroup:	7 years	11 years	7 years^b^	11 years
SLI	79.7 (8.5)	74.2 (6.4)	112.6 (10.7)	98.4 (12.5)
NSLI	77.0 (6.8)	72.8 (7.5)	94.2 (13.1)	63.5 (11.0)
LIQRes	83.5 (10.6)	94.1 (8.4)	101.3 (12.2)	68.4 (9.1)
Res	90.7 (11.0)	98.8 (11.6)	113.5 (11.6)	106.2 (15.7)

a TROG and PIQ are standard scores (mean  =  100, SD  =  15)

b PIQ at 7 years was measured using Ravens Coloured Progressive Matrices [38]

### Participants with Typical Development

A sample of 75 typically developing children, also aged 11 years, was recruited from primary schools in the Northwest of England. These children were attending regular school and had not received any learning support during their schooling (no speech therapy provision, special educational provision or support for hearing difficulties) and were native speakers of English. Thirty six were female (48%) and their average age was 11;0 (SD  =  3.5 months).

### Study Tasks

All children were administered the Children’s Test of Nonword Repetition (CNRep [39]) and a Past Tense morphology elicitation task (PT [40]). Scores for the participants are presented in Table 2. In addition, children were administered the CELF-R Recalling Sentences subtest (RecS [41]) which is a sentence repetition task.

**Table 2:** Memory and morphology scores for subgroups of children with a history of SLI and TD children.

	CNRep^a^		PT^a^	
SLI subgroup:	*M* (SD)	% correct	*M* (SD)	% correct
SLI	27.7 (6.5)	69	31.5 (12.2)	61
NSLI	24.1 (9.1)	60	29.0 (12.3)	56
LIQRes	27.4 (6.0)	69	38.6 (9.2)	74
Res	30.6 (7.5)	77	44.2 (6.8)	85
TD	36.4 (3.5)	91	46.8 (4.0)	90

a CNRep and PT results are raw scores (maximum 40 for CNRep, 52 for PT)

## Materials

### Children’s Test of Nonword Repetition (CNRep [39])

This test is designed to provide a measure of short-term phonological memory. It consists of 40 non-words which are presented in random order according to a standard phonetic pronunciation. The only departure from the procedure recommended by the authors of the test was that the stimuli were given using live voice with lips shielded to prevent lip-reading, rather than using a tape recording. The test was scored ‘on-line’ with each item judged as correctly or incorrectly repeated.

### Past Tense morphology elicitation task (PT [40])

This is designed to assess grammatical usage of verbs in past tense form. This task takes into consideration a number of features of verb tense complexity: verb class (regular vs. irregular), word frequency (high vs. low), stem final phonology (alveolar vs. non-alveolar) and neighbourhoods (friends vs. enemies). Neighbourhoods are defined as verb stems that share stem-final vowel or vowel-consonant phonemes. Some neighbours (e.g. *blow* and *grow*) are “friends” because they are neighbours in their stem and in their past tense form (i.e., *blow* →*blew* and *grow*→*grew*). In contrast, stem neighbours that do not remain neighbours in the past tense are “enemies”. For example, *blow*→*blew* and *sew*→*sewed* are enemies because one stem is suffixed, whereas the other undergoes a vowel change. The child is shown a picture depicting everyday activities while the assessor reads a sentence which has a missing word. The child is told to fill in the gap verbally to describe ‘what happened yesterday’ and the experimenter uses rising intonation to suggest that the sentence is incomplete. There are 52 verbs, both regular and irregular, that comprise the task. The child’s response was recorded and scored as correct or incorrect. Substitutions of a different main verb (with or without appropriate past tense inflection) were counted as incorrect as such substitutions are arguably related to features of verb tense complexity, for example, phonological features of items or item frequency [40].

### CELF-R Recalling Sentences subtest (RecS [41])

For this task children are given a sentence and asked to repeat it verbatim. Sentences become increasingly longer and more complex. Importantly, responses were scored according to test protocol, as this is the type of information which is readily available to clinicians and researchers working with children with SLI. The instructions for scoring focus on the number of errors made in each sentence: three points are awarded for a completely correct answer, two for a response with one error, one point for two or three errors and zero for four or more errors.

### Procedure

Each child was assessed separately by a researcher in a school setting and tests were administered in a quiet space outside the classroom.

## Results

Analysis of variance (Anova) was carried out followed by 10 post hoc tests in order to examine group differences between the five groups of children. Six correlational analyses (3 for SLI and 3 for TD) were undertaken to investigate relationships among CNrep, PT, and RecS. Two regression models were used to examine the contribution of the predictor variables (CNrep and PT) to performance in the recalling sentence task. Given the number of analyses and comparisons, significance in all cases was set at the .01 level. Effect sizes for multiple regression models were measured by Cohens f^2^ where 0.02 is regarded as a small, 0.15 a moderate and 0.35 a large effect size.

### Is Sentence Repetition Sensitive to Different Subgroups of Children with a History of SLI?

Children’s raw scores, by group, on CELF RecS are shown in table 3. Standard scores are also provided to aid with the interpretation of level of performance on the CELF RecS. All data analyses were carried out using raw scores. Initial data examination had revealed one high-scoring outlier in the NSLI group with a raw score of 65. This outlier was removed from all further analyses.

**Table 3:** Recalling sentences scores for subgroups of children with a history of SLI and TD children.

Group	Mean raw score (SD)	Raw score range	Mean standard score (SD)^a^
SLI subgroup:			
SLI	38.0 (9.9)	22–54	67.2 (4.0)
NSLI	36.8 (13.9)	3–59	67.4 (4.4)
LIQRes	49.0 (9.4)	26–67	73.5 (9.7)
Res	54.6 (10.4)	27–74	80.5 (13.4)
TD	63.0 (6.4)	45–75	(12.3)

a To aid interpretation, subtest scores were transformed to standard scores (mean = 100, SD = 15)

Anova on CELF RecS raw score showed a significant difference for group, (*F*(4, 266)  =  69.45, *p* < .001) with a large effect size (partial eta^2^  =  0.511). Equality of variance across the groups could not be assumed (Levene’s, *F*(4,266)  =  11.89, *p* < .001), therefore Tamhane’s T2 test was used for post hoc analysis, being robust where assumptions for the parametric Anova are not met. Post hoc tests showed significant differences between groups as follows; (SLI  =  NSLI) < (LIQRes  =  Res) < TD. CELF RecS raw score thus differentiates among children with continuing language problems (regardless of PIQ status), children with resolved language problems (again regardless of PIQ status), and TD children. However, all subgroups of children with a history of SLI, even those with resolved difficulties and currently performing within the normal range on receptive language, were still on average more than 1SD below the mean performance on the recalling sentences task.

### What is the Role of Memory Ability Versus Morphological Knowledge in Sentence Repetition in Middle Childhood?

Correlations between all relevant measures were examined in order to check for multicollinearity between potential predictor variables. Multicollinearity was not an issue for any of the models as evidenced by Table 4.

**Table 4:** Correlations between memory, morphology and recalling sentences raw scores for children with a history of SLI and TD children.

History of SLI:		
	PT	RecS
CNRep	.44***	.54***
PT	.	.64***
TD:		
	PT	RecS
CNRep	.42***	.43***
PT	.	.53***

*** *p* < .001

Transformations were applied to the raw scores of all of the variables of interest (RecS, CNRep and PT) to correct for the positively skewed distributions. The procedures that resulted in acceptable distributions (i.e. z-scores <1.96) were reflected square root transformations for the SLI RecS, CNRep and PT scores and TD RecS scores (each value subtracted from the maximum value + 1 and the square root taken) and reflected natural logarithm transformations for the TD CNRep and PT scores (each value subtracted from the maximum value + 1 and the logarithm to the base e of a number taken).

Multiple regression modelling was conducted with CELF RecS as the dependent variable. The predictor variables, CNRep and PT, were entered together in a single step. Separate analyses were carried out for children with a history of SLI and TD children (see table 5).

**Table 5:** Multiple regression analysis modelling memory and morphology as predictors of recalling sentences raw score in children with a history of SLI and TD children.

	History of SLI^a^			TD^b^		
Variable	B	SE B	β	B	SE B	β
CNRep	.40	.06	.36***	.29	.13	.21
PT	.42	.05	.48***	.53	.07	.45***

a *Adj R^2^*  =  .51

b *Adj R^2^*  =  .33

*** *p* < .001

The model for children with a history of SLI was significant, *F*(2, 186)  =  99.325, *p* < .001, and explained 51% of the variance in sentence repetition score (a large effect size, f^2^  =  1.07). Both memory (*p*<.001) and morphological abilities (*p*<.001) were significant explanatory factors in sentence repetition performance in children with a history of SLI at 11 years.

For TD children, the model was also significant, *F*(2, 72)  =  19.167, *p* < .001, and explained 33% of the variance in sentence repetition with a large effect size (f^2^  =  .53). However, morphological knowledge was the only significant predictor variable (*p*<.001). Specifically, phonological short term memory did not make a significant contribution to the prediction of sentence recall in 11-year-old children with typical development, whereas morphological abilities did.

## Discussion

This study provides new evidence for the key association between memory difficulties and language functioning in children with a history of SLI in middle childhood. The investigation highlights the contrast between these results and the findings with typically developing children for whom basic phonological memory processes do not appear to be as crucial at this stage of development. This study also extends previous research by demonstrating the value of sentence repetition as an indicator of language functioning in different subgroups of children with a history of SLI.

### Sentence Repetition and Subgroups of Children with a History of SLI

Consistent with previous research, sentence repetition was found to be sensitive as a marker of different subgroups of children with a history of SLI [3] [12]–[13] [15]. Children with a history of SLI in middle childhood were found to perform more poorly than typically developing peers regardless of whether they had current or resolved receptive language difficulties.

We also extend the research design of studies in this area by including children with varying levels of nonverbal cognitive skills. In this investigation, we provide new evidence that sentence repetition is problematic for children with current SLI with typical nonverbal skills as well as those with low nonverbal abilities. The pattern of their performance were similar, children with NSLI performing somewhat more poorly than children with SLI and typical nonverbal skills. These findings are consistent with previous research demonstrating similar, albeit poorer performance of children with NSLI versus SLI in areas such as speech of processing [42] and grammaticality judgements [43]. As would have been expected from previous research, difficulties with sentence repetition were also observed in children whose receptive language difficulties appeared resolved, who were performing within expectation for their age in standardised language assessments, and who had varying levels of nonverbal abilities. Together, the evidence suggests that, at least in middle childhood, level of nonverbal abilities is not a key factor in the performance of children in sentence repetition tasks. Our findings are consistent with evidence that nonverbal abilities do not correlate strongly with sentence repetition [3] [13] [28].

Sentence repetition produced a large effect size between subgroups of children with a history of SLI and their typically developing peers. On average, all children with a history of SLI, regardless of their verbal or non-verbal profiles were performing more than one standard deviation below the mean on the recalling sentence task. Such difficulties are striking. In addition, it is important to note that previous research with the Manchester Language Study has also directly compared sentence repetition with other markers of SLI. Of four potential markers investigated by Conti-Ramsden et al. [3] (the others being past tense provision, third person singular use and nonword repetition as measured by CNrep), sentence repetition showed the best combination of sensitivity and specificity in categorising children into SLI or TD groups. The findings of previous research and the results of the present study taken together suggest that sentence repetition is currently the best individual marker for SLI. This evidence suggests that future research examining the potential of recalling sentences as an endophenotypical marker of SLI is warranted.

### Factors Affecting Sentence Repetition Performance in Middle Childhood

It has been suggested that the effectiveness of sentence repetition in detecting SLI may well lie in its multifactorial nature, as the task is likely to involve both short and long term memory systems, as well as grammatical competence [3] [27]. What this study has clarified is that processes involved in language production are taxing of PSTM for individuals with a history of SLI in middle childhood in a way that does not appear to apply to TD children of the same age, at least within the context of the instruments and analyses used in this study. Memory abilities were a significant predictor of sentence repetition performance in individuals with a history of SLI.

In early childhood, PSTM appears to be implicated in language development. Gathercole and Baddeley [44] in their longitudinal study of typically developing 4 to 5 year old children demonstrated that PSTM as measured by nonword repetition accounted for a significant amount of variance in expressive language performance. Although comparisons across the present study and that of Gathercole and Baddeley [44] need to proceed with caution given the differences in the samples and instruments, these data are suggestive of developmental changes that may be taking place from early to middle childhood in children. A changing pattern of relative strength of predictors has also been seen in the nonword repetition of TD children [45].

What may these developmental processes entail? The model of memory proposed by Baddeley [9] suggests there is likely to be a reciprocal relationship between language attainment and PSTM. Thus, it may be the case that when confronted with a sentence repetition task, TD children in middle childhood can rely more heavily on their grammatical or other linguistic knowledge to process the sentences and recall them. The knowledge they have attained may support their performance. It may help them chunk sentences into fewer and more easily processable units or to map them onto more predictable structural representations, leading to a reduction in PSTM load. This does not appear to be the case for children with a history of SLI in middle childhood. Children with a history of SLI may not have developed strong enough representations to facilitate the task of sentence recall [26]. Thus, children with a history of SLI may not be able to rely as much on the predictability of sentence structures to chunk sentences, thus having to process or recall the sentence as a whole which is more demanding of PSTM. These suggestions highlight the need for more direct investigation of potential longitudinal relationships and developmental processes that may be affecting the language performance of children with and without SLI.

### Limitations and Clinical Implications

This investigation used a single task, CNrep, to assess the role of PSTM in performance in the recalling sentence task. It is important to note that in terms of cognitive loading, the CNrep places high demands on auditory speech perception and production and arguably lower demands on PSTM in comparison to other verbal memory tasks such as digit-span. Thus, the use of a single task to assess the role of PSTM loading in SLI is a limitation of this investigation. Further research is needed to establish whether the issue at stake in SLI is PSTM rather than more fundamental perceptual or production difficulties.

This study used the relatively coarse error scoring system as described in the test protocol for the recalling sentences task. Thus, the evidence presented is typical of that gained in clinical practice and is informative of factors involved in the general performance in this task; it does not allow us to identify the specific aspects of sentence repetition that are most vulnerable for children with SLI. More detailed error analyses are warranted. However, the sentence repetition task used here and most commonly used in practice, does not lend itself to this type of analysis because it is graded in sentence length and complexity and has a stopping rule resulting in children having different number of sentences of different complexities; not an adequate basis for error analysis.

This investigation focused on only two predictor tasks: nonword repetition and past tense affixation. Consideration of more complex models which examine other influencing variables and potential mediating factors such as complex working memory [46], other forms of lexical knowledge [19] or reading skills [47] would provide valuable information as to the nature of the relationships among these areas of functioning.

What this study does provide is further evidence of the value of sentence repetition as a marker of SLI. Sentence repetition abilities in middle childhood appear to be able to signal difficulties in different subgroups of children with SLI. This evidence points to the clinical utility of sentence repetition as a potentially informative assessment and/or screening instrument at this important stage of development when, in many education systems, children are about to make the transition from primary to secondary schooling.

Marker tasks which also provide insight into differences between affected and unaffected individuals in terms of the resources they use to perform a task, are valuable not only in developing our understanding of the disorder but for informing approaches to intervention. In this investigation, evidence is provided for the longer term sequelae of language difficulties and their likely impact on memory resources and how children process linguistic information in middle childhood. Should phonological working memory therefore be considered a target for intervention? A meta-analysis of current research on working memory training [48] suggests there are reliable short-term improvements in working memory skills after intervention with children and adults (in both typical and clinical samples). However, there is little evidence of transfer effects from working memory training to other skills, including verbal ability. Furthermore, there is good evidence that treatment involving direct training of language skills is effective [49]. The key clinical implication of these findings is that any memory intervention with children with SLI should be done *in conjunction with* explicit language training.

## References

[1] Newbury DF, Bishop DVM, Monaco AP (2005) Genetic influences on language impairment and phonological short-term memory. Trends Cogn Sci 9: 528–534. 16188486 10.1016/j.tics.2005.09.002

[2] Bishop DVM (2006) What causes specific language impairment in children? Curr Dir Psychol Sci 15: 217–221. 19009045 10.1111/j.1467-8721.2006.00439.x PMC2582396

[3] Conti-Ramsden G, Botting N, Faragher B (2001) Psycholinguistic markers for specific language impairment (SLI). J Child Psychol Psychiatry 42: 741–748. 11583246 10.1111/1469-7610.00770

[4] Leonard LB (1998) Children with specific language impairment. Cambridge Ma: MIT Press.

[5] van der Lely HKJ, Battell J (2003) WH-movement in children with grammatical SLI: A test of the RDDR hypothesis. Language 79: 153–181.

[6] Tallal P (2004) Opinion - Improving language and literacy is a matter of time. Nat Rev Neurosci 5: 721–728. 15322530 10.1038/nrn1499

[7] Leonard LB, Ellis Weismer S, Miller CA, Francis DJ, Tomblin JB, et al (2007) Speed of processing, working memory, and language impairment in children. J Speech Lang Hear Res 50: 408–428. 17463238 10.1044/1092-4388(2007/029)

[8] Baddeley AD (1986) Working memory. Oxford :Oxford University Press.

[9] Baddeley A (2003) Working memory and language: an overview. J Commun Disord 36: 189–208. 12742667 10.1016/s0021-9924(03)00019-4

[10] Archibald LMD, Gathercole SE (2006) Short-term and working memory in specific language impairment. Int J Lang Commun Disord 41: 675–693. 17079222 10.1080/13682820500442602

[11] Gathercole SE, Baddeley AD (1990) Phonological memory deficits in language-disordered children: Is there a causal connection? J Mem Lang 29: 336–360.

[12] Archibald LMD, Gathercole SE (2006) Nonword repetition: A comparison of tests. J Speech Lang Hear Res 49: 970–983. 17077209 10.1044/1092-4388(2006/070)

[13] Bishop DVM, North T, Donlan C (1996) Nonword repetition as a behavioural marker for inherited language impairment: Evidence from a twin study. J Child Psychol Psychiatry 37: 391–403. 8735439 10.1111/j.1469-7610.1996.tb01420.x

[14] Botting N, Conti-Ramsden G (2001) Non-word repetition and language development in children with specific language impairment (SLI). Int J Lang Commun Disord 36: 421–432. 11802495 10.1080/13682820110074971

[15] Conti-Ramsden G, Hesketh A (2003) Risk markers for SLI: a study of young language-learning children. Int J Lang Commun Disord 38: 251–263. 12851078 10.1080/1368282031000092339

[16] Ellis Weismer S, Tomblin JB, Zhang X, Buckwalter P, Chynoweth JG, et al (2000) Nonword repetition performance in school-age children with and without language impairment. J Speech Lang Hear Res 43: 865–878. 11386474 10.1044/jslhr.4304.865

[17] Bishop DVM, McDonald D, Bird S, Hayiou-Thomas ME (2009) Children who read words accurately despite language impairment: Who are they and how do they do it? Child Dev 80: 593–605. 19467013 10.1111/j.1467-8624.2009.01281.x PMC2805876

[18] Conti-Ramsden G, Durkin K (2007) Phonological short-term memory, language and literacy: developmental relationships in early adolescence in young people with SLI. J Child Psychol Psychiatry 48: 147–156. 17300553 10.1111/j.1469-7610.2006.01703.x

[19] Gathercole SE (2006) Nonword repetition and word learning: The nature of the relationship. Appl Psycholinguist 27: 513–543.

[20] Coady JA, Evans JL (2008) Uses and interpretations of non-word repetition in tasks in children with and without specific language impairments (SLI). Int J Lang Commun Disord 43: 1–40. 10.1080/13682820601116485 PMC5524521 18176883

[21] Bedore LM, Leonard LL (1998) Specific language impairment and grammatical morphology. A discriminant function analysis. J Speech Lang Hear Res 41: 1185–1192. 9771639 10.1044/jslhr.4105.1185

[22] Rice ML, Wexler K, Cleave PL (1995) Specific language impairment as a period of extended optional infinitive. J Speech Hear Res 38: 850–863. 7474978 10.1044/jshr.3804.850

[23] Leonard LB, Camerata SM, Brown B, Camerata MN (2004) Tense and agreement in the speech of children with specific language impairment: Patterns of generalization through intervention. J Speech Lang Hear Res 47: 1363–1379. 15842016 10.1044/1092-4388(2004/102)

[24] Archibald LMD, Joanisse MF (2009) On the sensitivity and specificity of nonword repetition and sentence recall to language and memory impairments in children. J Speech Lang Hear Res 52: 899–914. 19403945 10.1044/1092-4388(2009/08-0099)

[25] Marshall CM, Nation K (2003) Individual differences in semantic and structural errors in children's memory for sentences. Ed Child Psychol 20: 7–18.

[26] Riches NG, Loucas T, Baird G, Charman T, Simonoff E (2010) Sentence repetition in adolescents with specific language impairments and autism: an investigation of complex syntax. Int J Lang Commun Disord 45: 47–60. 19343567 10.3109/13682820802647676

[27] Poll GH, Betz SK, Miller CA (2010) Identification of clinical markers of specific language impairment in adults. J Speech Lang Hear Res 53: 414–429. 20360465 10.1044/1092-4388(2009/08-0016)

[28] Rice ML, Wexler K, Hershberger S (1998) Tense over time: The longitudinal course of tense acquisition in children with specific language impairment. J Speech Hear Res 47: 816–834. 10.1044/jslhr.4106.1412 9859895

[29] Lum JAG, Conti-Ramsden G, Page D, Ullman MT (2011) Working, declarative and procedural memory in specific language impairment. Cortex http://dx.doi.org/10.1016/j.cortex.2011.06.001 10.1016/j.cortex.2011.06.001 PMC3664921 21774923

[30] Rice ML, Tomblin JB, Hoffman L, Richman WA, Marquis J (2004) Grammatical tense deficits in children with SLI and nonspecific language impairment: Relationships with nonverbal IQ over time. J Speech Lang Hear Res 47: 816–834. 15324288 10.1044/1092-4388(2004/061)

[31] Rice ML, Wexler K (1996) Toward tense as a clinical marker of specific language impairment in English-speaking children. J Speech Lang Hear Res 39: 1239–1257. 10.1044/jshr.3906.1239 8959609

[32] Conti-Ramsden G, Botting N (1999) Characteristics of children attending language units in England: A national study of 7 year olds. Int J Lang Commun Disord 34: 359–366. 10884906 10.1080/136828299247333

[33] Conti-Ramsden G, Crutchley A, Botting N (1997) The extent to which psychometric tests differentiate subgroups of children with SLI. J Speech Lang Hear Res 40: 765–777. 9263942 10.1044/jslhr.4004.765

[34] Botting N (2005) Non-verbal cognitive development and language impairment. J Child Psychol Psychiatry 46: 317–326. 15755307 10.1111/j.1469-7610.2004.00355.x

[35] Conti-Ramsden G, St Clair MC, Pickles A, Durkin K (2012) Developmental trajectories of verbal and nonverbal skills in individuals with a history of SLI: From childhood to adolescence. J Speech Lang Hear Res. Advanced online publication. doi: 10.1044/1092–4388. 10.1044/1092-4388(2012/10-0182) 22562827

[36] Bishop DVM (1982) Test for Reception of Grammar. Manchester: University of Manchester.

[37] Wechsler D (1992) Wechsler Intelligence Scale for Children 3^rd^ edition. San Antonio, TX: The Psychological Corporation.

[38] Raven JC (1986) Coloured Progressive Matrices. London: H. K. Lewis.

[39] Gathercole SE, Baddeley AD (1996) The Children’s Test of Nonword Repetition. London :The Psychological Corporation.

[40] Marchman VA, Wulfeck B, Ellis Weismer S (1999) Morphological productivity in children with normal language and SLI: A study of the English past tense. J Speech Lang Hear Res 42: 206–219. 10025555 10.1044/jslhr.4201.206

[41] Semel E, Wiig E, Secord W (1994) Clinical Evaluation of Language Fundamentals-Revised. San Antonio, TX: The Psychological Corporation.

[42] Miller CA, Kail R, Leonard LB, Tomblin JB (2001) Speed of processing in children with specific language impairment. J Speech Lang Hear Res 44: 416–433. 11324662 10.1044/1092-4388(2001/034)

[43] Miller CA, Leonard LB, Finneran D (2008) Grammaticality judgments in adolescents with and without language impairment. Int J Lang Commun Disord 43: 346–360. 18446576 10.1080/13682820701546813 PMC2440708

[44] Gathercole SE, Baddeley AD (1989) Evaluation of the role of STM in the development of vocabulary in children: A longitudinal study. J Mem Lang 28: 200–213.

[45] Rispens J, Baker A (2012) Nonword repetition: the relative contributions of phonological short-term memory and phonological representations in children with language and reading impairment. J Speech Lang Hear Res 55: 683–694. 22223893 10.1044/1092-4388(2011/10-0263)

[46] Riches NG (2012) Sentence repetition in children with specific language impairment: an investigation of underlying mechanisms. Int J Lang Commun Disord, Early View.| Article first published online. :17 MAY 2012 DOI: 10.1111/j.1460-6984.2012.00158.x 10.1111/j.1460-6984.2012.00158.x 22938061

[47] Baird G, Slonims V, Simonoff E, Dworzynski K (2011) Impairment in non-word repetition: A marker for language impairment or reading impairment? Dev Med Child Neurol 53: 711–716. 21585364 10.1111/j.1469-8749.2011.03936.x

[48] Melby-Lervåg M, Hulme C (2012) Is working memory training effective? A meta-analytical review. Dev Psychol.Advance online publication. doi:10.1037/a0020228. 10.1037/a0028228 22612437

[49] Bowyer-Crane C, Snowling MJ, Duff FJ, Fieldsend E, Carroll JM, et al (2008) Improving early language and literacy skills: Differential effects of an oral language versus phonology with reading intervention. J Child Psychol Psychiatry 49: 422–432. 18081756 10.1111/j.1469-7610.2007.01849.x

